# Influence of rGO on the Crystallization Kinetics, Cytoxicity, and Electrical and Mechanical Properties of Poly (L-lactide-co-ε-caprolactone) Scaffolds

**DOI:** 10.3390/ma15217436

**Published:** 2022-10-23

**Authors:** Esperanza Díaz, Joseba León, Alberto Murillo-Marrodán, Sylvie Ribeiro, Senentxu Lanceros-Méndez

**Affiliations:** 1 Escuela de Ingeniería de Bilbao, Departamento de Ingeniería de Minera, Metalúrgica y Ciencia de Materiales, Universidad del País Vasco (UPV/EHU), 48920 Portugalete, Spain; 2 BcMaterials, Basque Centre for Materials, Applications and Nanostructures, (UPV/EHU) Science Park, 48940 Leioa, Spain; 3 Department of Mechanics, Design and Industrial Management, University of Deusto, Avda Universidades 24, 48007 Bilbao, Spain; 4 Centro de Física, Universidade do Minho, 4710-058 Braga, Portugal; 5 Centre of Molecular Environmental Biology (CBMA), Universidade do Minho, 4710-057 Braga, Portugal; 6 Ikerbasque Basque Foundation for Science, 48013 Bilbao, Spain

**Keywords:** PLCL/rGO, scaffolds, crystallization, electrical properties, mechanical properties, cytotoxicity

## Abstract

Biodegradable scaffolds of poly (L-lactide-co-ε-caprolactone) (PLCL) and reduced graphene oxide (rGO) were prepared by TIPS (thermally induced phase separation). The nonisothermal cold crystallization kinetics were investigated by differential scanning calorimetry (DSC) with various cooling rates. The experimental values indicate that nonisothermal crystallization improves with cooling rate, but the increasing rGO concentration delays crystallization at higher temperatures. The activation energies were calculated by the Kissinger equation; the values were very similar for PLCL and for its compounds with rGO. The electrical conductivity measurements show that the addition of rGO leads to a rapid transition from insulating to conductive scaffolds with a percolation value of ≈0.4 *w*/*w*. Mechanical compression tests show that the addition of rGO improves the mechanical properties of porous substrates. In addition, it is an anisotropic material, especially at compositions of 1% *w*/*w* of rGO. All of the samples with different rGO content up to 1% are cytotoxic for C2C12 myoblast cells.

## 1. Introduction

Originally, only materials capable of withstanding mechanical stresses equivalent to those of bone have been used in bone implants. Metallic materials, such as stainless steels and titanium alloys, met these requirements as bone fixation systems, but these require costly and risky surgical operations for their implementation and removal when they have fulfilled their purpose [1].

In the last decade, several biomaterials have been developed that can support the tissue regeneration process at the defect site and are subsequently resorbed and replaced with the newly generated tissue [2,3]. Many of these biomaterials belong to the family of aliphatic polyesters and are biodegradable and biocompatible, such as PLLA [4], PLG [5], PCL [6], and their copolymers PLGA [7] and PLCL [8]. These polymers allow relatively easy fabrication in the form of scaffolds in which the tissues to be repaired are implanted, attached, differentiated, and proliferated.

PLCL is a biodegradable copolymer of PLLA and PCL, belongs to the family of aliphatic polyesters, and its degradation rates can be manipulated by varying the ratio of the constituent polymers. It is approved by the United States Food and Drug Administration (U.S. FDA) for clinical application [9]. The addition of rGO to these biopolymers can significantly modify their electrical, physical, and mechanical properties [4,10]. Qi et al. [4] studied the effects of the addition of rGO in PLLA and found that there was an increase in the crystallinity of PLLA and an improvement in its piezoelectric properties. Seyedsalehi et al. [10] analysed the influence of the addition of rGo to PCL and found that it increases the strength, stiffness, and toughness of the scaffolds and produces mechanically resistant structures.

Many groups have studied the scaffolds of PLCL for their application in regenerative medicine [4,6,8,9], but none of the works have carried out an in-depth study of nonisothermal crystallization, conductivity, and cytotoxicity. This knowledge is essential because many of the polyesters’ processing operations (extrusion, injection molding) take place under nonisothermal conditions [11] and, during these processes, crystallization is too slow to develop significant crystallinity. The addition of some particles may, in same cases, accelerate the rate of crystallization.

In the literature, we can find some works in which the effect of the addition of a nucleating agent in the crystallization of PLLA and PCL is studied. The addition of these nucleating agents not only influences the crystallization, but also modifies the mechanical and electrical properties. Qui et al. [12] studied the effect on the crystallization of PLLA when talc is added. Zhou et al. [13] and Nejati et al. [14] analyzed the crystallization with nHA. Zhao et al. [15] studied the effect of the addition of CNTs on thermal, mechanical, and electrical properties.

In this work, the effect of rGO addition on crystallization, conductivity, cytotoxicity, mechanical, and electrical properties of PLCL scaffolds fabricated by thermally induced phase separation is studied.

## 2. Materials and Methods

Medical grade Poly(L-lactide-co-caprolactone) (70/30) was supplied from Purac Biomaterials (Purasorb PLC 7015, Amsterdam, The Netherlands). Gel permeation chromatography (GPC, Perkin Elmer 200, Triad Scientific, Manasquan, NJ, USA) was used to calculate the weight-average relative molecular weight: M_w_ = 130,489; M_n_ = 79759; and M_w_/M_n_ = 1.63.

### 2.1. Fabrication of Scaffolds

The fabrication of PLCL and PLCL/rGO composite scaffolds was performed by thermally induced phase separation (TIPS) and the freeze–drying technique and they were later dispersed by sonication.

**Figure 1:**
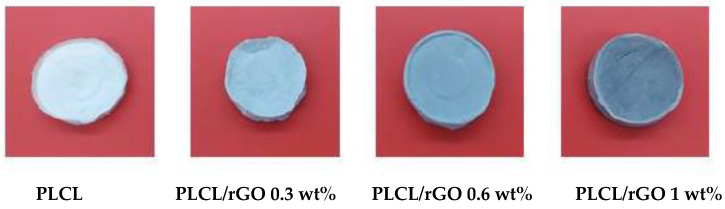
From left to right, samples of pure PLCL and PLCL/rGO at 0.3, 0.6, and 1 wt% rGO.

Porous scaffolds were prepared with 2.5% (*w/v*) of PLCL in 1,4-dioxane. The mixture was stirred at 50 °C for 2 h to obtain a homogeneous polymer solution. Reduced graphene oxide (rGO) was mixed with the solution of PLCL in a percentage of 0, 0.3, 0.6, and 1 wt% of the total polymer mass at room temperature. The resulting solutions were frozen and freeze-dried (LyoQuest of Telstar, Barcelona, Spain) at 60 °C and 0.5 mmHg for 10 days to remove the solvent. Scaffolds with a porosity of up to 90% were fabricated (see Figure 1).

### 2.2. Scanning Electron Microscopy (SEM)

The scaffolds were coated with gold, in a JEL Ion Sputter JFC-1100 (Amiron Machinery, Oxnard, CA, USA) at 1200 V and 5 mA. Scanning electron microscopy (SEM) (HITACHI S-3400N, Tokyo, Japan) was used to perform PLCL and PLCL/rGO micrographs.

### 2.3. Electrical Conductivity

The electrical conductivity (σ) at 37 °C was measured in a self-built thermostat chamber with a digital multimeter (SMU-236, Keithley Instruments, Cleveland, OH, USA). The electrical conductivities of scaffolds were obtained with a four-probe technique. Five specimens were electrically tested from each sample and their values were averaged.

### 2.4. Differential Scanning Calorimetry (DSC)

A DSC Q-200 TA Instruments calorimeter (Water, NC, USA) was used to determine the thermal properties of scaffolds. All tests were performed under nitrogen purge to prevent oxidation, and the weight of the samples varied between 4 and 6 mg. The samples were cooled to −40 °C until reaching the amorphous state and then heated to 200 °C and cooled again to −40 °C.

### 2.5. Mechanical Characterization

An Instron 5967 Universal Testing Machine (Instron, Norfolk County, MA, USA) was used to characterize the mechanical properties of porous supports following the ASTM D695 standard. For each composition, a total number of 10 cylindrical samples was extracted from the scaffold. Then, a set of uniaxial compression tests was performed at room temperature and a constant speed of 0.5 mm·min^−1^, using a load cell of 500 N. From the tests, the material elastic modulus and elastic limit were determined for each composition. To analyze the mechanical anisotropy, for each composition, half of the samples were tested in the axial direction and the other half in the radial direction of the scaffold. The results were obtained by averaging their values.

### 2.6. Cytotoxicity

For the cytotoxicity assays, sample sterilization is essential and, for that, membranes with 0.1 g·mL^−1^ were cut and sterilized by UV for 2 h before cell seeding (1 h each side). After that, the samples were washed five times with phosphate buffer saline (PBS) solution for 5 min to remove any residual solvent. This study is based in the indirect cytotoxicity evaluation of the samples in adaptation of the ISO 10993-5 standard test method.

The medium in contact with the samples (conditioned medium) was prepared by immersing the samples in a 24-well tissue culture polystyrene plate with DMEM (containing 4.5 g·L^−1^ glucose (Gibco, Madrid, Spain) supplemented with 10% fetal bovine serum (FBS, Biochrom, Cambridge, UK) and 1% penicillin/streptomycin (P/S, Biochrom, Cambridge, UK), at 37 °C in a 95% humidified air containing 5% CO_2_ and incubated for 24 h. Further, 20% of dimethylsulfoxide (DMSO, Sigma Aldrich, St. Louis, MO, USA) was used as a positive control and the cell culture medium was employed as a negative control.

In a 96-well tissue culture polystyrene plate, C2C12 cells were seeded at the density of 2 × 10^4^ cells·mL^−1^, at the same time, and incubated for 24 h. After 24 h, the culture medium from the 96-well tissue culture polystyrene plate was removed and the conditioned medium was added to the wells (100 µL). Afterwards, the cells were incubated for 72 h and, after each time, cell viability assessment was quantified with the (3-(4,5-dimethylthiazol-2-yl)-5-(3-carboxymethoxyphenyl)-2-(4-sulfophenyl)-2H-tetrazolium) (MTS) assay. At this time, the MTS reagent was added into each well (proportion 1/5 of MTS/DMEM medium) and incubated at 37 °C for 2 h. The absorbance was detected at 490 nm with a microplate reader.

All quantitative results were obtained from four replicates of the samples and controls and were analyzed as the average of viability ± standard deviation (SD).

The percentage of cell viability was calculated from the following formula:

(1) $$Cell\;viability\;\left(\%\right)=\frac{Absorbance\;of\;sample}{Absorbance\;of\;neegative\;control}\times 100$$

## 3. Results and Discussion

### 3.1. Morphology

The porosity and interconnectivity of the porous supports is a critical parameter as it allows cellular infiltration inside the material; exchange with the medium where it is implanted; and cellular adhesion, differentiation, and proliferation. It has been extensively reported that greater porosity improves osteogenesis [11,16,17,18].

**Figure 2:**
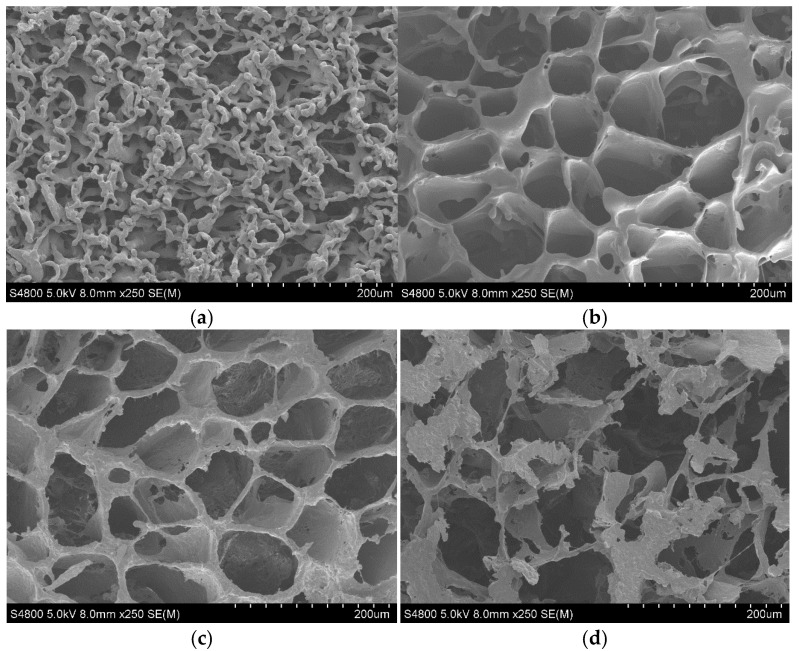
Representative SEM images of the surface morphology of PLCL composites. (**a**) PLCL × 250. (**b**) PLCL/rGO 0.3wt% × 250. (**c**) PLCL/rGO 0.6 wt% × 250. (**d**) PLCL/rGO 1wt% × 250.

In the micrographs shown in Figure 2, it can be observed how the pore size increases with the addition of rGO (20 µm for pure PLCL, 40–60 µm for samples with 0.3 and 0.6 wt% rGO, and between 80 and 100 µm for those with 1 wt%). The effect of rGO on the morphology of the scaffolds is striking, as it not only increases the pore size, but also makes the scaffolds more spheroidal and ordered in shape and the pore walls thicker. This thickness reaches a maximum value for the 0.3 wt% rGO samples and decreases when increasing the graphene concentration. These walls become more porous and less consistent with the addition of rGO. An extreme case is represented by the micrographs in Figure 2d corresponding to the samples with 1 wt% rGO, in which it can be seen that the tubular structure is lost, giving rise to a very porous but irregular structure without good definition of the porous walls. It seems that, from the morphological point of view, the most desirable structure would be achieved with the addition of 0.3–0.6 wt% rGO.

When larger amounts of rGO (≥1%) are added, the distribution of rGO throughout the polymer solution is less homogeneous, thus the structure of the scaffolds is more irregular. This behavior has also been found for PLLA scaffolds with MWCNTs [19], where the addition of nanotubes at concentrations of 1 and 5 wt% produces an ordered, uniform structure. However, at higher levels, the production and dispersion technique is ineffective, with the nanotubes tending to form aggregates.

### 3.2. Electical Conductivity Studies

Reduced graphene oxide has enormous potential as a reinforcing material in polymeric composites, as well as improving its electrical properties, which enables them to be used in a wide range of applications. The key challenge in the preparation of the scaffolds is to obtain a homogeneous dispersion of rGO, which is incompatible with most hydrophobic polymers in which it tends to pile up, thus preventing the formation of a continuous network for electron transport. Fabrication techniques are critical, with solution blending as well as in situ polymerization being the most commonly used [20,21,22].

**Figure 3:**
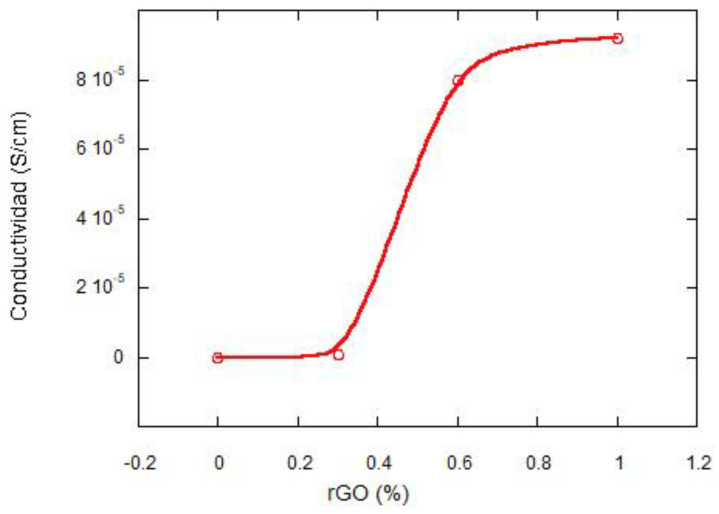
Variation of the electrical conductivity of the composites with increasing rGO concentration in %.

Figure 3 shows the variation in electrical conductivity as a function of rGO content. PLCL is an insulating polymer, but when rGO is added, the electrical conductivity increases significantly. The polymer undergoes a rapid transition from insulator to a more conductive material (typical percolation process). This is due to the formation of multiple conductive networks in the reduced graphene oxide. The so-called percolation threshold is the critical value at which an abrupt transition in conductivity occurs with a slight increase in the concentration of the conductive charge [20,21]. The value of the percolation threshold is low (0.4 wt%). This value is related to the properties of the composite materials; that is, high electrical conductivity of rGO (80 Scm^−1^), fairly uniform dispersion, and large size of the graphite sheets [22,23].

To investigate the dispersion of the reduced graphene oxide films, the conductivity of the scaffolds in the parallel and perpendicular direction to the pores was studied. The results can be seen in Table 1. It can be seen that the conductivities of the composites containing 0.6 wt% rGO are very similar in the parallel and perpendicular directions. It indicates a homogeneous dispersion of rGO and an isotropic behavior of the material. However, for concentrations of 1 wt% of rGO, very different values of conductivity are observed in the two directions studied, in terms of order of magnitude. At the latter concentration, the material is anisotropic, with different properties depending on the direction studied. Similar behavior has been found by Nariman Yousefi et al. [22] for polyurethane/rGO nanocomposites at compositions of 2 to 5 wt%. It can be concluded that, when an electric field is applied, electrons can flow into the scaffolds through interconnected pore channels, with a percolation threshold value of 0.4 wt%.

### 3.3. Thermal Properties

Crystalline polymers can crystallize between the glass transition temperature (Tg) and melting temperature (Tm). Depending on what the initial state is, the crystallization process can be classified into two categories; one would be melt crystallization, where the polymer must initially remain at a temperature above their Tm, and the other would be cold crystallization, which means that the initial state is the amorphous state and the polymer samples must remain at a temperature lower than their Tg^25^. In this work, the crystallization of pure PLCL and its composites with 0.3, 0.6, and 1% rGO from the amorphous state was studied.

**Figure 4:**
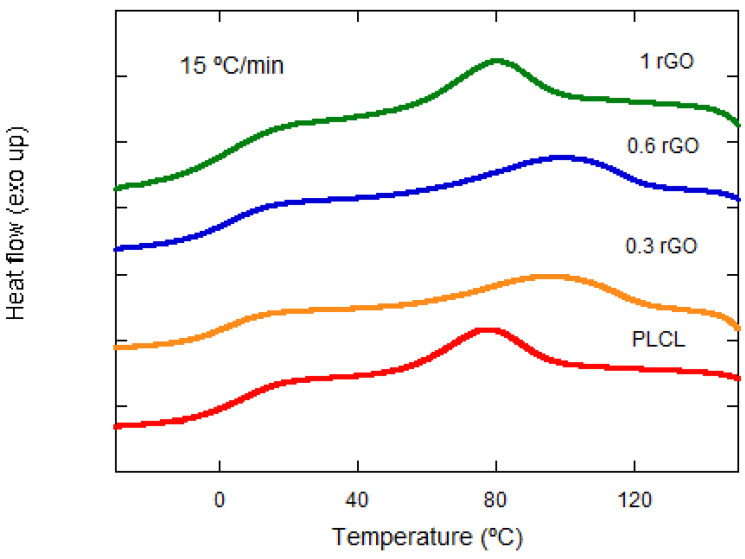
Nonisothermal cold crystallization behavior of neat PLCL and its composites at 15 °C·min^−1^ from the amorphous state (in the vertical axis, the units are missing).

The samples were subjected to different cooling rates of 5, 10, 15, and 20 °C·min^−1^. They were first cooled down to −40 °C and then heated up to 200 °C and again cooled down to −40 °C (at different cooling rates). First, the effect of rGO on the crystallization process was studied. Figure 4 shows how, for the same cooling rate (15 °C·min^−1^), the crystallization temperature reaches higher values as the rGO content increases, except for the sample with 1 wt% rGO.

The effect of the cooling rate on nonisothermal cold crystallization is observed in Figure 4, with the crystallization peak becoming wider and moving to lower temperatures with the increasing cooling rate for the same rGO composition. This behavior is observed for all of the compositions studied.

**Figure 5:**
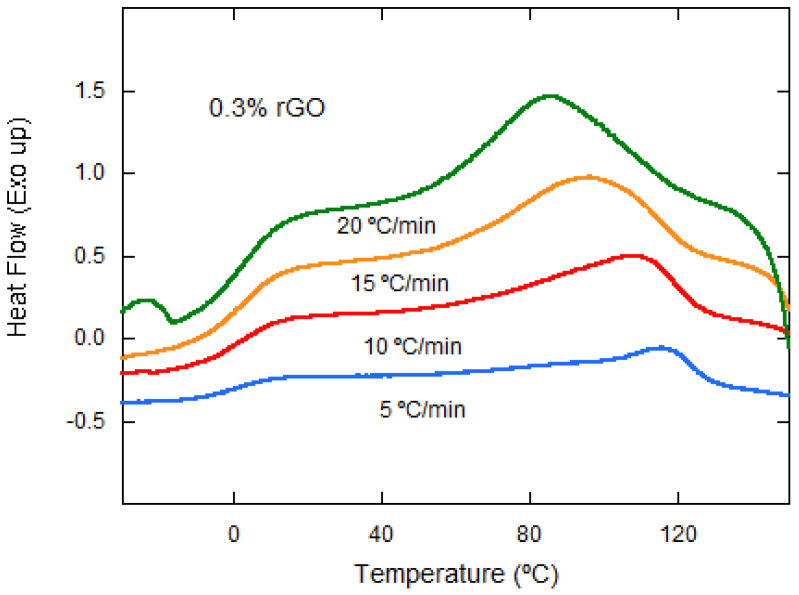
Nonisothermal cold crystallization behavior of PLCL/rGO 0.3 wt% at different cooling rates.

According to the obtained experimental results, Table 2 shows how, at all of the tested speeds and for the same rGO composition, the temperature of the nonisothermal crystallization peak (Tp) decreases as the cooling speed increases. The evaluation of the influence of the rGO concentration at all of the tested speeds (Figure 5) shows that the Tp increases with the addition of rGO, regardless of the amount incorporated.

The experimental results obtained indicate that the rGO concentration and cooling rate strongly affect the crystallization process. That is, the higher the cooling rate, the lower the Tp and, when rGO is added, it increases. Therefore, the nonisothermal crystallization process improves with the cooling rate, but the increase of rGO slows down the crystallization process at higher temperatures.

**Figure 6:**
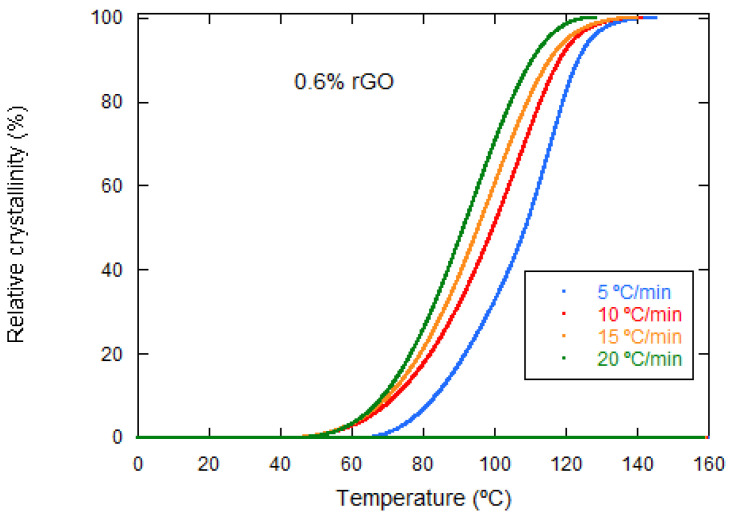
Relative crystallinity versus temperature of PLCL/rGO 0.6 wt% at different cooling rates.

The integration of the exothermic peaks versus temperature during nonisothermal crystallization allows to obtain the relative crystallinity versus crystallization temperature for cooling rates of 5–20 °C·min^−1^. In Figure 6, it is observed how the crystallization temperature decreases with the increasing cooling rate. This behavior has been observed for all of the samples studied and indicates that a higher rate does not slow down the crystallization process, but rather has the opposite effect. In fact, the opposite behavior has been reported in the literature, such as Zhao et al. [15] and Wang et al. [15,24,25], although they were reporting on MWCNTs and GO, respectively. In other words, it seems that the type of reinforcement used has a strong influence on the crystallization process.

**Figure 7:**
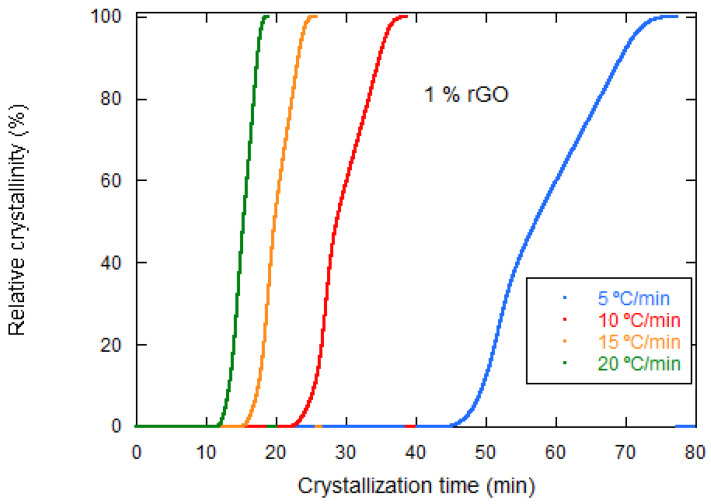
Relative crystallinity versus crystallization time for PLCL/rGO 1 wt% at different cooling rates.

The relationship between the crystallization time *t* and the corresponding temperature *T* during the nonisothermal cold crystallization process is related to the following expression [26]:

(2) $$t=\frac{Tc-T}{\Phi }$$

where *Tc* is the temperature at crystallization time, *To* is the initial crystallization temperature, and ***Φ*** is the cooling rate. The integration of the (exothermic) crystallization peaks during the nonisothermal crystallization process allows to obtain the relative crystallinity versus crystallization time for cooling rates of 5, 10, 15, and 20 °C·min^−1^ (Figure 7). It is observed that the crystallization time increases with the decreasing cooling rate. For example, the PLCL/rGO 1 wt% samples needed 7 min to complete the crystallization process at a fast cooling rate of 20 °C·min^−1^. However, 30, 19, and 11 min were used for rates of 5, 10, and 15 °C·min^−1^, respectively.

The mean crystallization time (t_1/2_) is the time required for the support to acquire 50% of the total crystallinity and can be obtained directly from the values of the relative crystallinity versus crystallization time plots. These values are very important to discuss the nonisothermal cold crystallization rate of pure PLCL and PLCL/rGO studied at different cooling rates. Table 1 shows all t_1/2_ values for pure PLCL and its three composites with rGO at different cooling rates. It is observed that t_1/2_ decreases with the increasing cooling rate for both pure PLCL and its rGO composites, suggesting that the rate of general nonisothermal crystallization becomes faster with the increasing cooling rate. It is shown that the t_1/2_ values are higher in the scaffolds made with rGO than those of pure PLCL at a given cooling rate. This trend suggests that the nonisothermal cold crystallization behavior of PLCL is not enhanced by the presence of rGO owing to the nucleation effect. This behavior is very different from that found by Wang et al. [26] in their studies with PLLA/GO, but it should be noted that they are dealing with GO and not rGO, as in the present paper; in addition, a copolymer is used.

**Figure 8:**
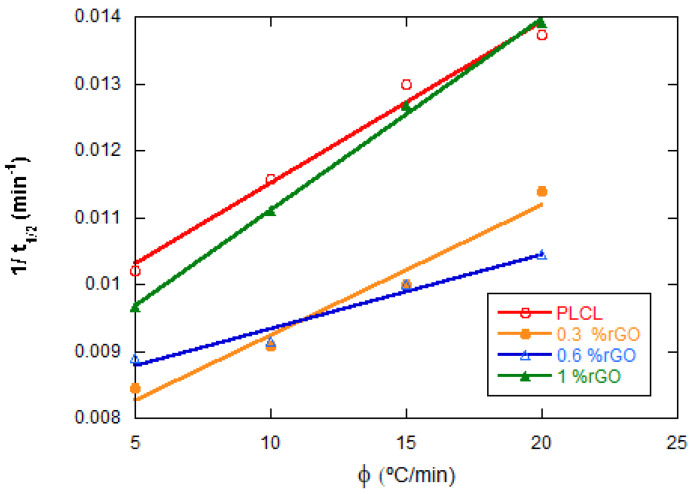
Effect of rGO content on the PLCL crystallization rate for the four compositions studied. Crystallization rate parameter.

To quantitatively evaluate the effect of rGO on the crystallization rate, the CPR parameter [27,28,29,30], which is the crystallization rate parameter of the polymer, has been proposed. The CRP can be calculated from the slope of the lines representing 1/t_1/2_ versus the cooling rate (see Figure 8). A higher slope value would indicate a higher crystallization rate. The values obtained were 0.991 for PLCL and 0.985, 0.980, and 0.999 for its compounds with 0.3, 0.6, and 1 wt% of rGO, respectively. The values obtained for CRP are very similar for all composites.

**Figure 9:**
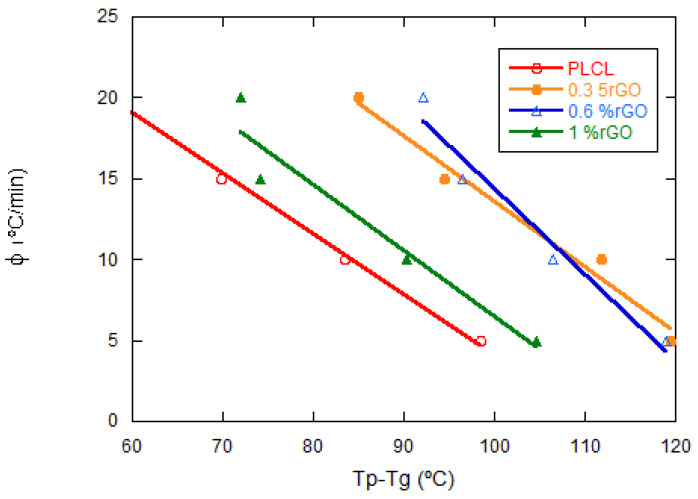
Effect of rGO content on the PLCL crystallization rate for the four compositions studied. Crystallization rate coefficient.

Khanna et al. [31] proposed to compare the crystallization rate of different polymer systems using a crystallization rate coefficient (CRC), which represents a change in the cooling rate required to produce a 1 °C change in the supercooling of the melting polymer. The CRC can be used as a guide to rank the polymer on a single scale of crystallization rates. CRC values should be higher for faster crystallizing systems. The CRC value could be determined from the slope of the linear plot of the cooling versus Tm–Tp, where Tm and Tp represent the melting point and maximum nonisothermal crystallization temperature, respectively. In this work, the crystallization behavior of PLCL and its composites with the rGO has been studied from the melt state; therefore, the CRC determination has been modified using Tp–Tg instead of Tm–Tp, where Tp and Tg are the maximum nonisothermal crystallization temperature and the glass transition temperature, respectively, representing the change in the cooling rate required to cause a 1 °C change in the supercooling of the melt phase of the polymer (see Figure 9). The CRC coefficient of the PLCL sample was 0.999 and that of its composites with rGO 0.3, 0.6, and 1 wt% were 0.996, 0.977, and 0.991, respectively.

Further investigation has been carried out using Kissinger’s method, calculating the activation energies of pure PLCL and its compounds to elucidate the effect of rGO nucleation on nonisothermal cold crystallization [32]:

(3) $$\frac{d(\ln\left(\frac{\Phi }{Tp^{2}}\right)}{d\;\left(\frac{1}{Tp}\right)}=-\frac{\Delta E}{R}$$

**Figure 10:**
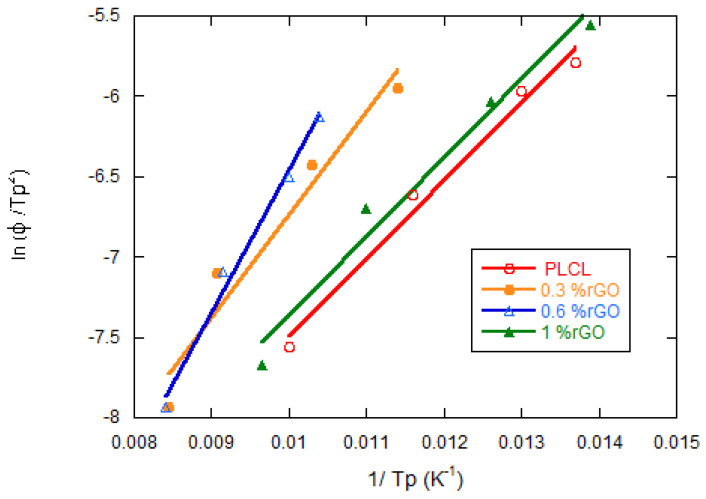
Kissinger plots of PLCL and PLCL/rGO composites for the estimation of crystallization activation energy.

In this equation, Δ*E* is the activation energy, *Tp* is the temperature of the crystallization peak, and *R* is the universal gas constant. By plotting d(ln (*Φ*/*Tp^2^*)) versus 1/*Tp*, four straight lines are obtained, one for each composition, from whose slope multiplied by *R* we obtain the activation energy of nonisothermal crystallization. Figure 10 shows that the four lines have very similar slope values.

The obtained values are 8.24 for pure PLCL and 8.09, 8.25, and 8.24 kJ·mol^−1^ for the scaffolds with 0.3, 0.6, and 1 wt% rGO, respectively. It can be seen how the activation energy is very low for all samples and hardly changes with the rGO content. From the results obtained using Kissinger’s method for the activation energy, it can be stated that the addition of rGO in the PLCL matrix does not increase the activation energy, which indicates that rGO does not act as a physical barrier that delays crystallization, although it does not favor it either, as we have seen previously. Wang et al. [25] found that the addition of GO reinforcements to PLLA increases the activation energy by GO acting as a crystallization nucleant.

### 3.4. Cytotoxicity

All of the samples were also evaluated with respect to their cytotoxicity to understand their possible potential for biomedical applications. The cytotoxicity of PLCL and PLCL-rGO samples was evaluated, according to the ISO standard 10993-5, with myoblast cells (C2C12), and the results obtained after 72 h are shown in Figure 9.

Cytotoxicity tests are very important to determine the potential biomedical use of the materials.

**Figure 11:**
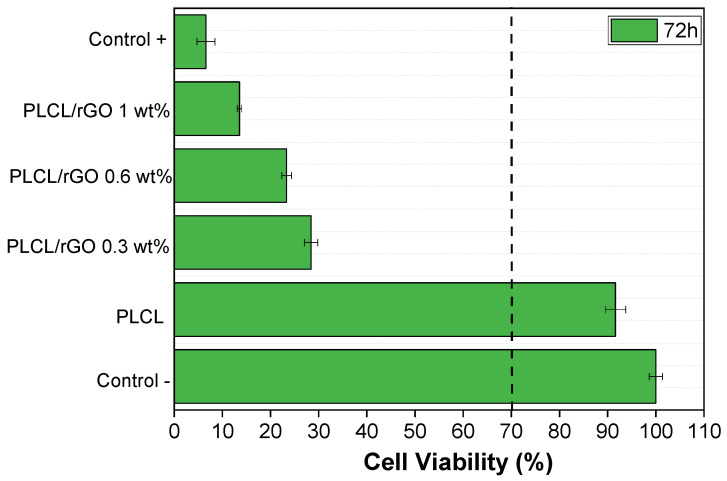
Cell viability of C2C12 cells in contact with the conditioned media exposed with PLCL with different % of rGO up to 72 h.

Figure 11 shows that, after 72 h, the only sample that is not cytotoxic is pristine PLCL, confirming the results of the literature [33]. All composites with different rGO contents up to 1% in the PLCL matrix are cytotoxic for C2C12 cells, demonstrating that they cannot be used for other cell culture assays. This is because of the fact that all samples present cell viability values below 70% compared with the control (according to the ISO standard 109935-5, the samples are considered cytotoxic when the cells have a cell viability decrease higher than 30%). Other studies with similar percentages of rGO particles, but with a PLLA matrix, have been reported as non-cytotoxic [34]. This may lead one to consider that certain bonds are formed between the polymeric matrix and the particles, being harmful for the cells, or that there is a release of some harmful component, remaining from the processing, to the medium. The addition of rGO is thus believed to produce pores with a morphology in which it is more difficult to remove the solvent, which is toxic.

### 3.5. Mechanical Properties

Porous polymeric supports present limitations in their mechanical resistance, mainly due to the presence of these pores, which constitute the weakest area of the material. In the case of the present study, it was chosen to add a polymeric coating with rGO reinforcement, which acts as a bonding agent, increases cohesion, and improves the mechanical resistance and hardness of the porous supports. As a result, a biomaterial composed of a polymeric matrix and rGO reinforcement is obtained that can be suitable for use in bone tissue regeneration. In order to analyze its behavior in the elastic regime in which its use is conceived, the most relevant mechanical parameters studied were the modulus of elasticity and the mechanical resistance of the scaffold.

The temperature of the material has an effect on its modulus of elasticity and mechanical strength. The higher the temperature, the lower the modulus of elasticity and the lower the mechanical strength. On the other hand, the strain rate can also alter the mechanical properties of the material; its decrease has similar effects to the increase in temperature [35]. The effects of temperature and strain rate are not considered in this study. Therefore, all tests were performed at room temperature and at a constant speed of 0.5 mm·min^−1^.

**Figure 12:**
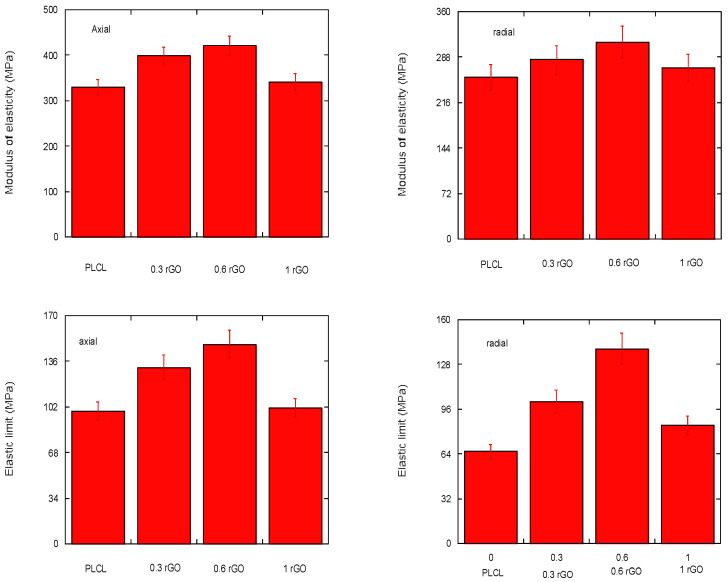
Compressive modulus of elasticity and elastic limit under compression, in the axial and radial direction, for scaffolds made with 0, 0.3, 0.6, and 1% rGO.

Owing to the nature of the porous scaffold, it was expected that it might show anisotropy in its mechanical properties. Therefore, of the 10 samples obtained for each composition, half are tested in the axial direction and the other half in the radial direction. In Figure 12,the values obtained for both the compressive modulus of elasticity and compressive yield strength in the axial and radial test directions can be observed.

From the depicted results, it can be pointed out that scaffolds show differences in their mechanical response depending on their tested orientation, as expected. This confirms the material mechanical anisotropy, which can be explained by its porous structure. In addition, if the rGO reinforcement is not evenly distributed in the composite, it can also affect the mechanical response of the material. Therefore, the anisotropy is linked to both the uneven reinforcement distribution and structural porosity of the material. Moreover, two factors that strongly influence the mechanical properties of the scaffolds are pore size and wall thickness [36]. These depend on both the temperature and time of the freeze–drying process and on the amount of rGO.

In this study, the lyophilization process is similar for all of the tested specimens. However, noticeable differences can be appreciated depending on the rGO content. From Figure 12, it can be stated that, the higher the rGO content, the higher the load required to deform the material, except for the 1% wt PLCL/rGO scaffolds. Similarly, when the required stress for the yielding of the material is higher, the rGO content is higher and, again, there is the exception of the PLCL/rGO 1 wt% scaffold. This is aligned with the results previously shown for scaffold morphology. In the scaffold PLCL/rGO 1 wt%, the tubular structure is lost and its rGO distribution throughout the polymer solution is less homogeneous. This leads to a very porous but irregular structure without good definition of the porous walls. Thus, it is linked to a worse mechanical response and higher mechanical anisotropy.

## 4. Conclusions

Nonisothermal cold cristallization, cytotoxicity, and electrical and mechanical properties of biodegradable PLCL/rGO scaffolds were studied in this work. From the crystallization study, it can be concluded that, at the different speeds tested and for the same composition, the temperature of the nonisothermal crystallization peak (Tp) decreases as the cooling rate increases. If the influence of the composition of the porous supports is analyzed, it can be seen how the Tp increases as the amount of rGO increases, except for the PLCL/rGO 1 wt% scaffolds. The values of the activation energy obtained by the Kissinger equation indicate that rGO does not act as a nucleanting agent in the crystallization process. When an electric field is applied, a high percentage of electrons can flow through the interconnected channels of the scaffolds, as long as the sample is above the percolation threshold value. All composite scaffolds show a reduction in cell viability of more than 30%, thus being considered cytotoxic. The analysis of the mechanical properties indicated that the porous supports synthesized with 1% *w*/*w* were anisotropic owing to the irregular rGO distribution.

## Figures and Tables

**Table 1:** Electrical conductivity of PLCL/rGO scaffolds in the direction perpendicular to the pores and in the parallel direction.

rGO Content (wt%)	Conductivity (S·cm^−1^) Perpendicular to Pores	Conductivity (S·cm^−1^) Parallel to Pores
0	1 × 10^−12^	1 × 10^−12^
0.3	2 × 10^−6^	5 × 10^−7^
0.6	3.5 × 10^−5^	8 × 10^−5^
1	7 × 10^−8^	9.2 × 10^−5^

**Table 2:** Summary of relevant thermal parameters for neat PLCL and its nanocomposites: cooling rate (Φ), nonisothermal peak temperature (Tp), crystallization enthalpy (ΔHc), and nonisothermal crystallization half-time (t_1/2_).

Samples	Φ (°C/min)	Tp (°C)	ΔHc (J/g)	t_1/2_ (min)
Neat PLCL	5	98.13	20.10	10.15
	10	86.31	19.50	5.26
	15	76.71	13.52	4.25
	20	72.77	5.27	3.46
PLCL/0.3% rGO	5	118.38	8.47	13.45
	10	110.10	12.53	6.60
	15	96.46	13.60	4.42
	20	87.54	12.79	3.36
PLCL/0.6% rGO	5	118.36	6.55	13.92
	10	109.38	13.44	6.87
	15	99.87	15.51	4.63
	20	95.71	12.27	3.45
PLCL/1% rGO	5	103.57	18.95	13.17
	10	89.91	19.10	6.74
	15	78.92	13.56	4.72
	20	71.86	5.591	3.72
